# Free fatty acids and triglyceride change in the gallbladder bile of gallstone patients with pancreaticobiliary reflux

**DOI:** 10.1186/s12944-021-01527-4

**Published:** 2021-08-31

**Authors:** Yukai Xiang, Xiangyu Kong, Cheng Zhang, Chuanqi He, Jingli Cai, Ruiqi Lu, Bosen Zhang, Liu Lu, Yulong Yang

**Affiliations:** 1 Center of Gallbladder Disease, Shanghai East Hospital, Institute of Gallstone Disease, School of Medicine, Tongji University, 200092 Shanghai, China; 2 Department of Laboratory Medicine, Shanghai East Hospital, School of Medicine, Tongji University, 200092 Shanghai, China

**Keywords:** Pancreaticobiliary reflux, Gallstone formation, Gallbladder bile, Free Fatty Acids, Triglyceride

## Abstract

**Background:**

Pancreaticobiliary reflux (PBR) causes chronic inflammation of the gallbladder mucosa and changes in the bile components, which are known to promote gallstone formation. This study aimed to investigate the bile biochemistry changes in gallstone patients with PBR and provide new clues for research on the involvement of PBR in gallstone formation.

**Methods:**

Patients undergoing surgery for gallstones between December 2020 and May 2021 were eligible for inclusion. The bile biochemistry (including amylase, lipase, triglyceride, cholesterol, free fatty acids [FFAs], alanine aminotransferase [ALT], aspartate aminotransferase [AST], alkaline phosphatase [ALP], and γ-glutamyl transferase [γ-GT]) of the included gallstone patients was analysed to determine correlations with PBR.

**Results:**

In this study, 144 gallstone patients who underwent surgery were enrolled. Overall, 15.97 % of the patients had an increased bile amylase level, which was associated with older age and significantly higher bile levels of ALP, lipase, triglyceride, and FFAs. Positive correlations were observed between amylase and lipase, triglyceride, FFAs levels in the gallbladder bile. However, the bile levels of triglyceride, FFAs, and lipase were positively correlated with each other only in the PBR group and showed no significant correlation in the control (N) group. In addition, elevated bile FFAs levels were found to be an independent risk factor for gallbladder wall thickening.

**Conclusions:**

In conclusion, PBR-induced increase in FFAs and triglyceride in the gallbladder bile is a cause of gallstone formation, and an increase in bile ALP suggests the presence of cholestasis in PBR.

## Background

Gallstones represent a common gastrointestinal disease affecting 10–20 % of adults worldwide [1]. A number of risk factors have been reported to be associated with gallstones, including metabolic abnormalities, genetic and environmental risk factors, as well as pancreaticobiliary reflux (PBR), which is a newly discovered risk factor [1, 2]. Traditionally, PBR has been reported to occur in pancreaticobiliary maljunction (PBM), which is a congenital malformation where the pancreatic and biliary ducts unite to form a long common channel outside the duodenal wall where the sphincter of Oddi (SO) is absent [3]. Thus, the pancreaticobiliary junction is not directly controlled by the SO, resulting in the reflux of pancreatic enzymes into the biliary tract and gallbladder [3]. Pancreatic enzymes, especially amylase, are generally elevated in the bile within the biliary tract and gallbladder of patients with PBM [3–6]. However, there is evidence that PBR can also occur in patients with a morphologically normal pancreaticobiliary junction but without PBM [7–12].

The mechanism of gallstone formation due to PBR is still unclear, and may be related to the hydrolysis of phospholipids and induction of chronic inflammation of gallbladder mucosa. Pancreatic enzymes that are refluxed into the bile duct are easily activated [12, 13]. For example, phospholipase A2 (PLA2), which is activated in the biliary tract, hydrolyses phosphatidylcholine (PC), resulting in a lower PC content in the bile [6, 13]. Cholesterol, which is slightly soluble in aqueous media, is soluble in bile due to a mixture of bile salts and phospholipids [14]. At a lower PC concentration, cholesterol-lecithin vesicles may become unstable and release cholesterol crystals, which may contribute to gallstone formation [2, 15, 16]. In addition, the derivatives of PC hydrolysis by PLA2, such as lysophosphatidylcholine (lysoPC), have been reported to cause biliary epithelial cell damage, leading to chronic mucosal inflammation, hyperplasia, and metaplasia, which are responsible for gallstone formation, gallbladder cancer and biliary tract cancer [6, 12, 13, 17, 18]. In animal models and gallstone patients, gallstone formation is preceded by inflammatory changes in the gallbladder wall, including increased gallbladder wall thickness [1, 19]. Lee et al. even found that inflammation is a necessary factor for cholesterol gallstone formation in a model of gallstone formation in prairie dogs [20].

A comprehensive exploration of the interaction between PBR and gallbladder bile is needed to deepen the current understanding of the pathogenesis of gallstones. This study analysed the levels of amylase, lipase, triglyceride, free fatty acids (FFAs), cholesterol, alanine aminotransferase (ALT), aspartate aminotransferase (AST), alkaline phosphatase (ALP), and γ-glutamyl transferase (γ-GT) in the gallbladder bile of gallstone patients, with the aim of providing new evidence for the correlation between PBR and gallstone formation.

## Patients and methods

**Fig. 1:**
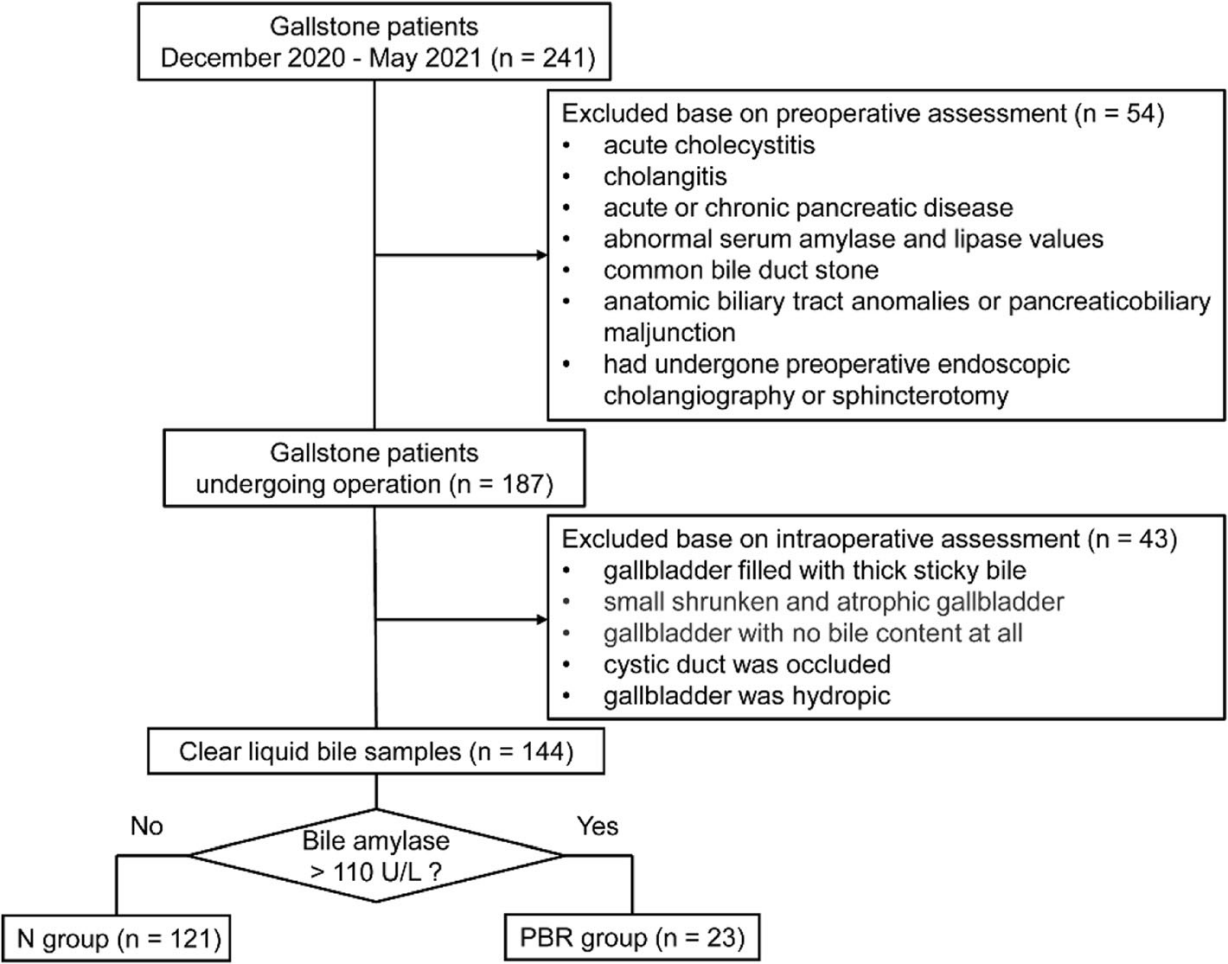
Consort flowsheet outlining the selection of the study cohort

According to the inclusion criteria shown in Fig. 1, this study prospectively and consecutively included 144 patients who underwent surgery for gallstones between December 2020 and May 2021. Blood samples were obtained the day before surgery and immediately delivered to the laboratory to measure amylase and other biochemical parameters. During the operation, at least 5 ml bile was obtained from the gallbladder using a syringe, stored in a sterile tube at 4 °C, and sent to the laboratory on the next day. All samples were processed and measured by laboratory technicians who were not informed of the study and source of samples using a Roche Cobas c702 (Roche Diagnostics, Basel, Switzerland); the normal value of serum amylase was 30–110 U/L. Various methods can be employed to identify PBR, and this study applied a widely accepted approach that involves extraction of the bile directly from the gallbladder during the operation followed by measurement of bile amylase [10–12, 21, 22]. Although there is no consensus on normal amylase levels in either the gallbladder or biliary tract, this study, like most investigations, used normal plasma amylase levels as a reference, and bile amylase levels higher than the normal plasma amylase levels as indicative of PBR [10–12, 22–24]. In patients with PBR, the ultrasonographic finding of gallbladder wall thickness > 3 mm was considered to be mucosal inflammation and hyperplasia [25]. Thus, this study evaluated mucosal inflammation by measuring the gallbladder wall thickness, and gallbladder wall thickness > 3 mm was identified as gallbladder wall thickening.

Data were represented as medians (25th – 75th percentiles). All statistical analyses were performed using the SPSS Statistics version 24 (IBM Corporation). The t-test and chi-square test were used for comparing continuous and categorical variables, respectively. Correlation analysis was performed using the Pearson correlation test. Univariate and multivariate logistic regression analyses were performed to identify the risk factors for gallbladder wall thickening. Statistical significance was set at *P*-value < 0.05.

## Results

In this study, 144 patients who underwent surgery for gallstones between December 2020 and May 2021 included; 23 patients were identified as the PBR group, whereas 121 patients were the control (N) group (Fig. 1). The median age was 56 years (50–65) in the PBR group and 45 years (35–60) in the N group (*P* = 0.010) (Table 1). Gender, body mass index (BMI), hypertension, hypercholesterolemia, hypertriglyceridemia, diabetes history, abdominal pain, stone number or size, complicated with gallbladder polyps or gallbladder adenomyomatosis, and gallbladder wall thickening showed no clear correlation with the incidence of PBR.

**Table 1:** Clinical characteristics of gallstone patients with or without PBR

Variables	N group (*n* = 121)	PBR group (*n* = 23)	*P*-value
Gender (M/F)	46/75	10/13	NS
Age (years), median (Q1 - Q3)	45 (35–60)	56 (50–65)	0.010
BMI (kg*m^− 2^), median (Q1 - Q3)	24.05 (22.22–25.72)	23.21 (21.65–25.63)	NS
Hypertension, n (%)	25.62 %	21.74 %	NS
hypercholesterolemia, n (%)	1.65 %	8.70 %	NS
hypertriglyceridemia, n (%)	9.92 %	26.09 %	NS
Diabetes history, n (%)	6.61 %	4.35 %	NS
Abdominal pain, n (%)	64.46 %	73.91 %	NS
No. of stone (Single/multiple)	35/86	10/13	NS
Diameter of stone (≤ 1 cm/>1 cm)	73/48	15/8	NS
Complicated with GBP, n (%)	24.79 %	13.04 %	NS
Complicated with GA, n (%)	12.40 %	13.04 %	NS
GWT > 3 mm, n (%)	29.75 %	47.82 %	NS

Data are represented as the median (25th − 75th percentiles) or percentage

*N* control, *PBR* pancreaticobiliary reflux, *M *male, *F* female, *BMI* body mass index, *GBP* gallbladder polyps, *GA* gallbladder adenomyomatosis, *GWT* Gallbladder wall thickness, *NS* no significance

**Fig. 2:**
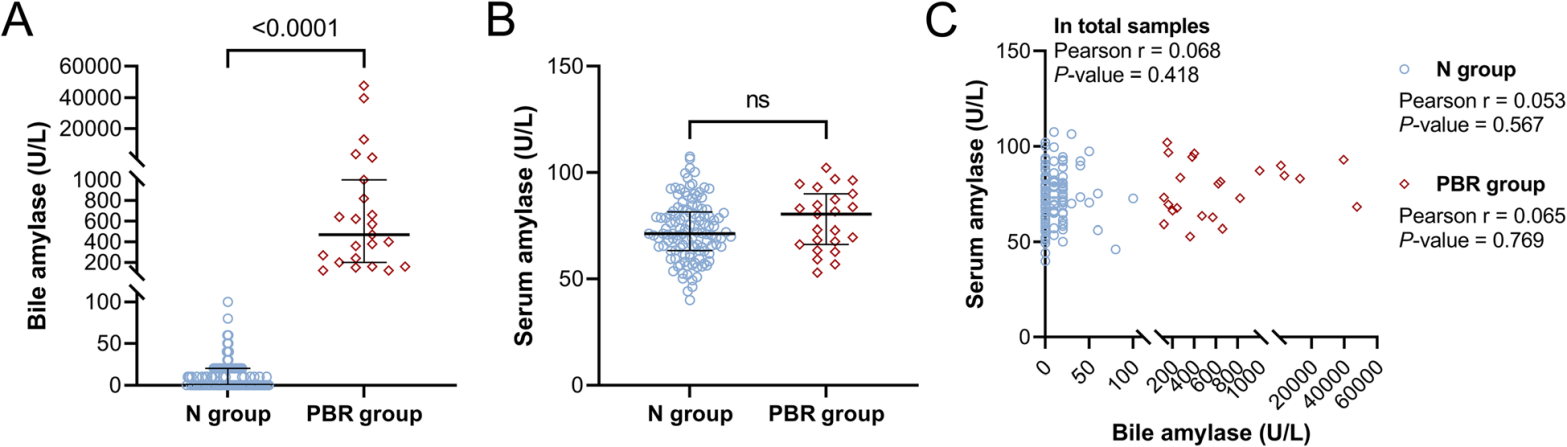
Amylase levels in the gallbladder bile and serum in gallstone patients with PBR. **A** Amylase levels in the gallbladder bile of gallstone patients. **B** Amylase levels in the serum of gallstone patients. **C** The correlation between the levels of bile amylase and serum amylase. The ns means no significance

**Fig. 3:**
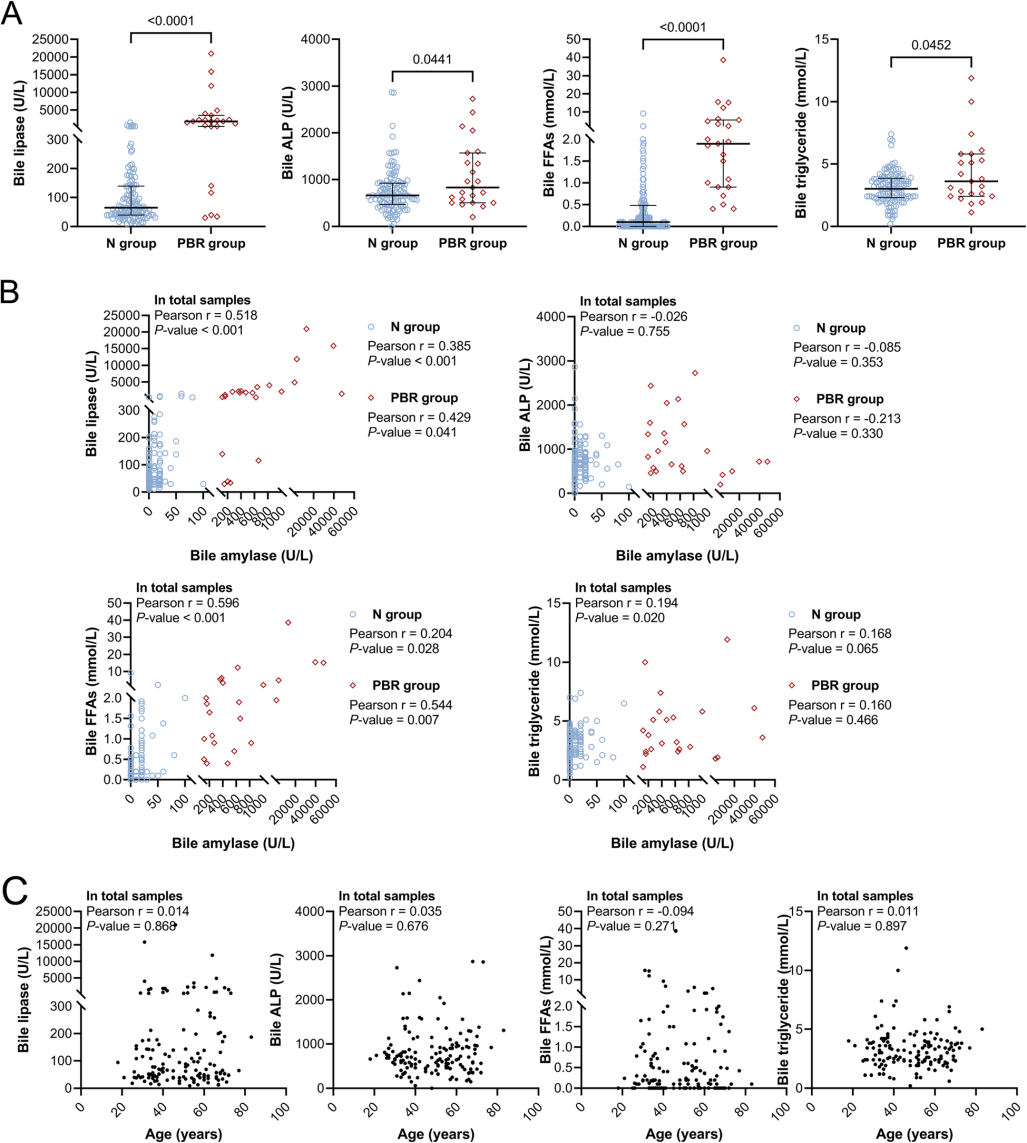
Bile levels of lipase, ALP, FFAs and triglyceride in gallstone patients with PBR. **A** The levels of lipase, ALP, FFAs and triglyceride in the gallbladder bile of gallstone patients. **B** The correlation between the bile levels of amylase and lipase, ALP, FFAs, triglyceride. **C** The correlation between the age and the bile levels of lipase, ALP, FFAs, triglyceride

The median bile amylase level in the PBR group was 470 U/L (200–1000); it was significantly higher than that in the N group, which was 0 U/L (0–20) (Fig. 2A). However, the serum amylase levels in the two groups were not significantly different (Fig. 2B). In addition, there was no correlation between the levels of bile amylase and serum amylase (Fig. 2C). Table 2; Fig. 3A show that the bile levels of lipase, FFAs, triglyceride and ALP were significantly higher in the PBR group than in the N group. However, there were no significant differences in the bile levels of triglyceride, ALT, AST, and γ-GT between the N and PBR groups (Table 2). In addition, the bile levels of lipase, triglyceride, and FFAs were significantly and positively correlated with bile amylase, except for bile ALP. Among these, bile lipase and bile FFAs were positively correlated with bile amylase in both the N and PBR groups (Fig. 3B). However, they did not show any correlation with age (Fig. 3C).

**Table 2:** Biochemical characteristics of gallbladder bile in gallstone patients with or without PBR

Variables	N group (*n* = 121)	PBR group (*n* = 23)	*P*-value
Lipase (U/L)	64.00 (39.00–138.90)	1769.50 (309.00–3463.50)	< 0.001
ALT (U/L)	4 (0–21)	4 (0–8)	NS
AST (U/L)	86 (52–198)	102 (44–348)	NS
ALP (U/L)	660 (460–920)	830 (500–1570)	0.044
γ-GT (U/L)	2600 (1360–5145)	4380 (1420–8920)	NS
Cholesterol (mmol/L)	11.70 (9.10–14.95)	11.20 (8.20–13.80)	NS
Triglyceride (mmol/L)	3.00 (2.30–3.85)	3.60 (2.40–5.80)	0.046
FFAs (mmol/L)	0.10 (0.00–0.48)	1.90 (0.90–5.52)	< 0.001

Data are expressed as the median (25th − 75th percentiles)

*N* control, *PBR* pancreaticobiliary reflux, *ALT *alanine aminotransferase, *AST* aspartate aminotransferase, *ALP* alkaline phosphatase, *γ-GT* γ-glutamyl transferase, *FFAs* free fatty acids, *NS* no significance

**Fig. 4:**
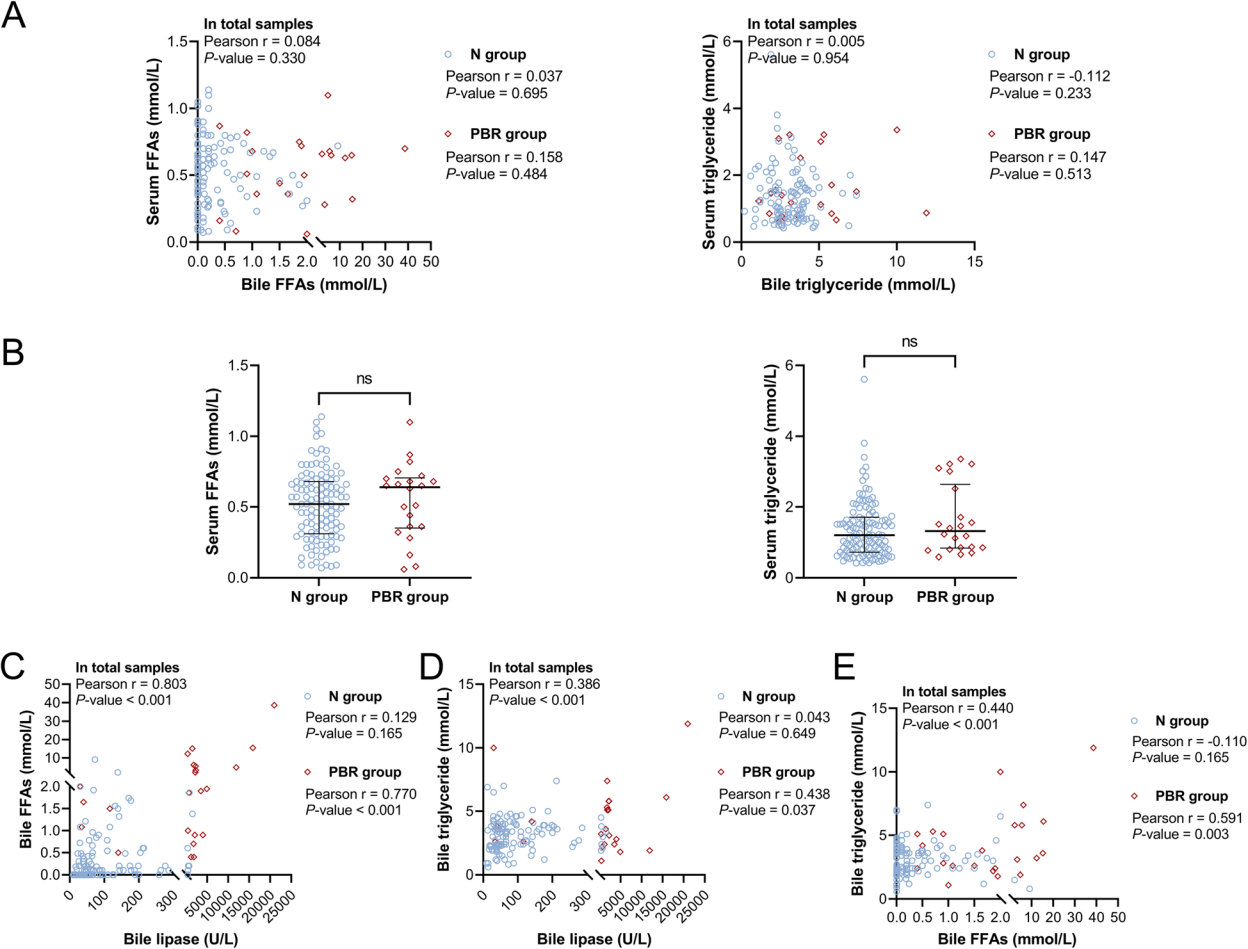
Correlation among the bile levels of FFAs, triglyceride and lipase in gallstone patients. **A** The correlation between the levels of FFAs or triglyceride in bile and serum. **B** The levels of FFAs and triglyceride in the serum of gallstone patients. **C** The correlation among the bile levels of FFAs, triglyceride and lipase in gallstone patients. The ns means no significance

Further analysis revealed no significant correlation between the levels of FFAs or triglyceride in the bile and serum, and no significant difference in the serum levels of FFAs and triglyceride between the N and PBR groups (Fig. 4A and B). However, there was a significant positive correlation among lipase, FFAs, and triglyceride levels in the bile of gallstone patients (Fig. 4C). Interestingly, this correlation was present in the PBR group only and was absent in the N group. Finally, the possible risk factors for gallbladder wall thickening were analysed using univariable logistic regression (Method: Enter); the selected variables (*P* < 0.2) were then subjected to multivariate logistic regression (Forward LR). Similar to gallbladder adenomyosis, the bile FFAs were independently associated with gallbladder wall thickening (Table 3).

**Table 3:** Univariable and multivariable logistic analyses for gallbladder wall thickening

Variables	Univariable analysis		Multivariable analysis	
	HR (95% CI)	***P***-value	HR (95% CI)	***P***-value
Gender (Male/female)	1.04 (0.51 – 2.12)	0.919		
Age	1.02 (0.99 – 1.04)	0.189	-	NS
BMI	1.07 (0.96 – 1.20)	0.243		
Hypertension (No/yes)	1.99 (0.91 – 4.33)	0.084	-	NS
Hypercholesterolemia (No/yes)	6.55 (0.66 – 64.71)	0.108	-	NS
Hypertriglyceridemia (No/yes)	1.04 (0.36 – 2.96)	0.946		
Diabetes (No/yes)	2.77 (0.71 – 10.83)	0.144	-	NS
Abdominal pain (No/yes)	1.33 (0.63 – 2.82)	0.455		
Stone number (Single/multiple)	0.96 (0.45 – 2.02)	0.905		
Diameter of stone (≤1cm/>1cm)	0.74 (0.36 – 1.52)	0.407		
Complicated with GBP (No/yes)	0.72 (0.31 – 1.70)	0.455		
Complicated with GA (No/yes)	2.32 (0.85 – 6.29)	0.100	3.26 (1.15 – 9.25)	0.027
PBR (No/yes)	2.16 (0.87 – 5.36)	0.095	-	NS
Bile lipase	1.00 (1.00 – 1.00)	0.099	-	NS
Bile ALP	1.00 (1.00 – 1.00)	0.516		
Bile triglyceride	1.09 (0.88 – 1.34)	0.447		
Bile FFAs	1.18 (1.00 – 1.40)	0.053	1.20 (1.01 – 1.43)	0.036

*BMI* body mass index, *GBP* gallbladder polyps, *GA* gallbladder adenomyomatosis, *PBR* pancreaticobiliary reflux, *ALP* alkaline phosphatase, *FFAs* free fatty acids, *HR* Hazard ratio, *CI* confidence interval, *NS* no significance

## Discussion

The reflux of pancreatic enzymes into the biliary tract and gallbladder, which is induced by PBR, causes chronic inflammation of gallbladder mucosa and changes in the bile components, eventually resulting in the formation of gallstones [7, 24, 26, 27]. Chronic inflammatory changes in the gallbladder mucosa are often accompanied by reduced gallbladder motility and modified bile transportation, absorption, and secretion, which could induce lithogenic bile [19]. For example, an increased secretion of gallbladder mucus in the chronically inflamed gallbladder shortens the nucleation time, contributes to the development of cholestasis and microlithiasis, ultimately leading to the formation of gallstones [13, 27, 28]. Furthermore, elevated levels of PLA2, an active pancreatic enzyme, which can hydrolyse PC, can cause the cholesterol-lecithin vesicles to become unstable and release cholesterol crystals, leading to gallstone formation [13, 15, 16]. This study investigated the effects of PBR on bile biochemistry in gallstone patients, and provides a deeper insight into how PBR induces gallstone formation.

Bile amylase is considered a useful biochemical marker for pancreatic enzyme reflux into the bile duct and gallbladder [10–12, 21–24]. Under physiological conditions, the level of bile amylase obtained from the biliary tract using endoscopic retrograde cholangiopancreatography is regarded as equivalent to that obtained from the gallbladder [22]. In this study, gallbladder bile amylase levels were determined to demonstrate the prevalence of PBR in patients with gallstones [8, 29]. Several investigators have studied the prevalence of high bile amylase levels in patients with gallstones [8, 29]. Horaguchi et al. reported a high incidence (15.4 %) of increased biliary amylase in 26 patients with cholecystolithiasis [8], and Sakamoto et al. found that the prevalence of elevated gallbladder amylase levels in 87 cholelithiasis patients was 12.6 % [29]. In this study, the incidence of increased bile amylase levels in gallstone patients (15.97 %) was similar to that in the above-mentioned studies. However, compared to the N group, patients with PBR were likely to be older (median, 56 vs. 45 years; *P* = 0.010). However, the bile levels of lipase, triglyceride, ALP and FFAs were not affected by age, as they were not correlated with age.

Based on bile biochemistry analysis, it was found that the levels of triglyceride, FFAs, and ALP in the bile were elevated in the PBR group, although they did not differ significantly in the blood between the two groups. In addition, triglyceride and FFAs levels in the bile had no correlation with the blood levels, but were significant positively correlated with amylase and lipase levels in the bile. However, the correlations among the lipase, triglyceride, and FFAs levels in bile were present in the PBR group only, and were absent in the N group. This indicates that the increase in triglyceride and FFAs in the bile does not come from the blood, but is caused by changes in the bile components induced by the reflux of pancreatic fluid.

Significant elevations in ALP are mainly seen in diseases associated with intra- or extrahepatic cholestasis [30]. However, PBR occurs frequently in patients with a long common channel at the junction of the pancreatic and biliary ducts [31, 32]. A relatively long common channel (≥ 5 mm) and papillitis were identified as significant predictive factors for high biliary amylase levels [33]. The results of the present study showed that the bile ALP was increased in the PBR group and showed no correlation with the bile amylase, suggesting that the increase in ALP was not caused by pancreatic reflux but due to cholestasis present in patients with PBR, which may be related to the relative obstruction of bile outflow caused by a long common channel and papillitis [34].

Consistent with previous reports, FFAs were elevated in the bile of PBR patients, and were independently associated with gallbladder wall thickening, indicating the presence of gallbladder mucosal inflammation and hyperplasia [13, 17]. FFAs in bile have always been thought to concentrate from hepatic bile and be produced by the phospholipases, especially phospholipase A2, which hydrolyses phospholipids [13, 35]. However, this study assumed that at least some of them were derivatives of lipases, such as hydrolyzing triglyceride. These FFAs in high concentrations could directly cause epithelial cell damage and result in mucus hypersecretion, gallbladder hypomotility, and gallstone nucleation [36–39]. In normal individuals, the gallbladder epithelium is capable of absorbing lipids from bile [13, 40, 41]. Thus, gallbladder epithelial injury could reduce the ability to absorb bile lipids, leading to the accumulation of triglyceride in bile. There is evidence that impaired gallbladder motility in individuals with hypertriglyceridemia is due to reduced sensitivity to cholecystokinin, which is relevant for gallbladder motility [42–44]. Furthermore, when the gallbladder motility and functioning is impaired, it could lead to cholestasis and even further increase in the level of FFAs in the gallbladder bile [35].

### Comparisons with other studies and what does the current work add to the existing knowledge

In this study, an elevation in bile amylase was found in 15.97 % of the gallstone patients, indicating that PBR is a common phenomenon in patients with gallstones. Thus, its roles in gallstone formation need to be identified urgently. Currently, with regard to the changes in the bile composition in patients with PBR, most studies have focused on elevated lysoPC, which is thought to cause damage to the mucosal barrier injuring the cell membrane as a major factor of biliary inflammation, gallstone formation, and carcinogenesis [6, 12, 13, 17]. Although the elevated FFAs in the bile of patients with PBR has also been reported, it is only considered one of the derivatives of PC hydrolysis by PLA2 to lysoPC [17]. However, its value in gallstone formation in patients with PBR is little recognized. Studies have reported the significance of FFAs in gallstone formation; yet, the idea that the elevation of FFAs could be caused by PBR has gone unnoticed [18, 36–39]. An investigation of the link between elevated FFAs in bile and the gallstones caused by PBR hasn’t been conducted. In this study, apart from the elevated FFAs in the gallbladder bile of PBR patients, it was also identified for the first time that the increases in the triglyceride and ALP, and the bile FFAs levels were independent risk factors for gallbladder wall thickening. It was assumed that at least some FFAs were derivatives of lipases, such as hydrolyzing triglyceride, and that the elevated levels of FFAs and triglyceride in the gallbladder bile are the cause of gallstone formation. These results deepen the current understanding of the source of FFAs for gallstones and the important roles of FFAs in gallbladder formation in patients with PBR. Based on the present study, further investigations will be carried out on the role and mechanism of FFAs in PBR caused gallstones, gallbladder cancer and biliary tract cancer, with a view to bringing new strategies for the diagnosis as well as treatment of PBR.

### Strengths and limitations of this study

This study has several strengths. First, the results revealed that FFAs, triglyceride, and ALP were elevated in the gallbladder bile of gallstone individuals with PBR, and that FFAs levels were strongly correlated with the levels of amylase, lipase, and triglyceride. Furthermore, FFAs were independently associated with gallbladder wall thickening, indicating that FFAs may be an important factor in PBR to promote gallstone formation through chronic inflammation. These results deepen the current understanding of the pathogenesis of gallstones in individuals with PBR.

The main limitation of the current study was that it was conducted in a relatively small population based in a single centre over a short period of time, which may have affected the ability to detect statistically significant changes in the gallbladder bile of individuals with PBR and gallstones. In addition, differences in the color of the gallbladder bile in individuals may have influences on the results measured via a colorimetric method.

## Conclusions

**Fig. 5:**
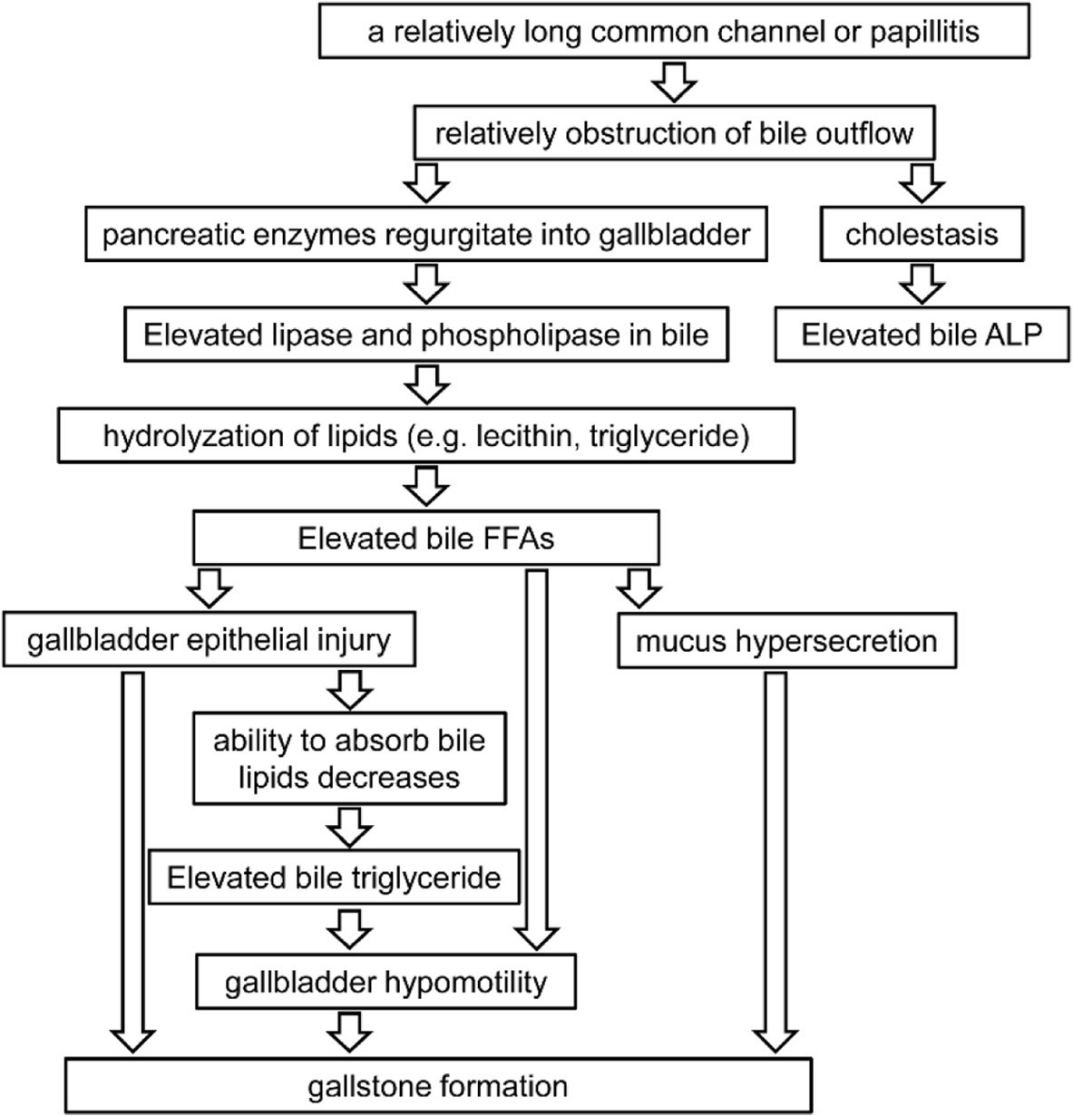
Speculative mechanism of gallstone formation caused by elevated FFAs and triglyceride in the gallbladder bile of patients with PBR

The above results suggest that the increase in FFAs and triglyceride in the gallbladder bile, which is induced by PBR, causes the formation of gallstones, and an increase in the bile ALP suggests the presence of cholestasis in PBR. The potential mechanism is shown in Fig. 5; due to PBR, pancreatic enzymes regurgitate into the gallbladder such that the levels of phospholipase and lipase increase in the gallbladder bile, causing the hydrolyzation of lipids (e.g., lecithin, triglyceride) to produce large amounts of FFAs, further inducing gallbladder epithelial injury, mucus hypersecretion, gallbladder hypomotility as well as gallstone nucleation and formation. Following gallbladder epithelial injury, its ability to absorb lipids decreases, resulting in an increase in triglyceride, which is thought to be associated with poor gallbladder motility, contributing to the stasis of refluxed pancreatic juice in the gallbladder and promotion of gallstone formation. These results strengthen the current understanding of the mechanism of gallstone formation in patients with PBR, and further investigations are needed to identify the types of fatty acids that change in patients with PBR and their roles in gallstone formation. In the future, the treatments that interrupt PBR may be an effective therapeutic and preventive measure for gallstones in individuals with PBR, such as endoscopic sphincterotomy to shorten the relative long common channel at the junction of the pancreatic and biliary ducts [45].

## Data Availability

The data used to support this study are available from the corresponding author upon reasonable request.

## Abbreviations

PBR: Pancreaticobiliary reflux
PBM: Pancreaticobiliary maljunction
SO: Sphincter of Oddi
PLA2: phospholipase A2
lysoPC: Lysophosphatidylcholine
FFAs: Free fatty acids
ALT: Alanine aminotransferase
AST: Aspartate aminotransferase
ALP: Alkaline phosphatase
γ-GT: γ-glutamyl transferase
N: Control
BMI: Body mass index
